# Carvacrol

**Published:** 1874-10

**Authors:** E. A. Bogue

**Affiliations:** New York.


### ARTICLE VIII.

### *Carvacrol.*

BY E. A. BOGUE, M. D., NEW YORK.

This substance, when first introduced to the profession, was of such a disagreeable odor, and so unpleasant to handle, that it was almost impossible to use it. It is now, however, thanks to the efforts of Dr. Sage, being furnished in an almost unobjectionable form; and as it has one or two qualities certainly, that commend it to use, it will be well to notice any new ones, as they may be discovered. One of these qualities, which was, I think, first noticed by Dr. A. L. Northrop, is its property of dissolving gutta percha; especially when the latter is warmed. Hence, if Carvacrol has been put into a sensitive cavity, a thin layer of gutta percha, or Hill's stopping, may be spread evenly over the bottom, without drying the cavity out; thus making use of the solvent property of the Carvacrol, while it is doing its work in allaying sensitiveness; or the cavity may be completely filled, and the burnisher moistened with the surplus Carvacrol, will finish the filling much more smoothly than usual, if finished in the ordinary way. For the insertion of root fillings, in cases where gutta percha is desirable, and for a great deal of the patching that requires to be done, where pivot teeth have been worn for a good many years, and where oxy-chlo. zinc is not admissible, Carvacrol proves a very valuable adjunct.—*Johnston's Dental Miscellany.*

